# Severe Acute Respiratory Syndrome Coronavirus-2 Pneumonia Presenting Concomitantly With Purpura Fulminans: A Case Report

**DOI:** 10.7759/cureus.21188

**Published:** 2022-01-13

**Authors:** Giulio Ciprian

**Affiliations:** 1 Internal Medicine, Roger Williams Medical Center, Providence, USA

**Keywords:** covid and purpura fulminans, coronavirus disease (covid-19), pulmonary critical care, covid-19, acute infectious purpura fulminans

## Abstract

Severe acute respiratory syndrome coronavirus-2 (SARS-CoV-2) is a novel coronavirus that has been extensively described in its most common presentations. Its pathogenetic process is poorly understood, although it is theorized that endothelial damage and inflammation play a central role. Its prothrombotic nature affects multiple organs, including lungs, kidneys, and the central nervous system. Rarer are cutaneous presentations that can be triggered or displayed concomitantly with COVID-19. Purpura fulminans is a life-threatening syndrome that results in skin thrombosis and hemorrhagic infarction. While its association is explicit in critically ill patients with sepsis, few or rare cases have been described to be linked with COVID-19. In this report, we present a case of a critically ill patient with COVID-19 who showed signs of purpura fulminans while in the intensive care unit.

## Introduction

The severe acute respiratory syndrome coronavirus-2 (SARS-CoV-2) is a novel coronavirus that has rapidly spread reaching pandemic proportions. Respiratory compromise affects many individuals infected with the virus causing acute respiratory distress syndrome (ARDS). However, patients with COVID-19 are also at higher risk for hypercoagulable events such as deep venous thrombosis (DVT), pulmonary embolism (PE), and, in rare instances, involvement of other organs. Most of these hypercoagulable events are traceable to venous thromboembolism (VTE) as the source. Unlike disseminated intravascular coagulation (DIC) where the majority of clinical findings are related to bleeding, COVID-19 has a higher association with thrombosis. The pathophysiology behind the COVID-19 hypercoagulable state is incompletely understood. Hyperviscosity was one of the observed mechanisms [1]. Some experts postulate endothelial injury and venous stasis as the contributing etiology to these VTE events in the settings of microvascular inflammation and acute systemic inflammatory response led by a cytokines storm such as interleukin (IL)-6 and other acute phase reactants [2]. There have been rare case reports of purpura fulminans associated with COVID-19 infection [3-5]. In this condition, protein C deficiency or severe acute sepsis is accountable for most of the cases, although an idiopathic etiology is also common. Regardless of the underlying cause, the mechanism is analog to protein C deficiency. In critically ill patients with COVID-19 hospitalized in intensive care unit (ICU), widespread inflammatory response and endothelial injury lead to systemic coagulation activation and depletion of coagulation factors and platelets. Protein C not only acts as an anticoagulant but also modulates the inflammatory response by downregulating proinflammatory and proapoptotic mediators, therefore, stabilizing the cell's barrier functions [6]. In the early stages, capillary blockage by blood clots leads to capillary congestion, while in later stages, irreversible ischemic changes on the endothelium are responsible for gangrenous necrosis. Herein, we present an interesting and rare case of a young female who was intubated and admitted to ICU for acute hypoxic respiratory failure secondary to SARS-CoV-2 virus, expressing signs of purpura fulminans during her hospitalization in the ICU.

## Case presentation

**Figure 1:**
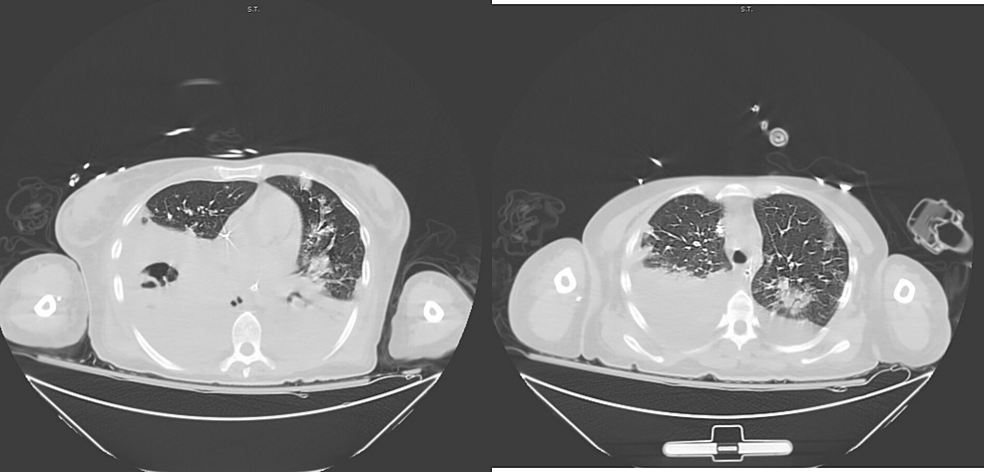
Chest CT. Left: Chest CT suggestive of necrotizing pneumonia with possible abscess on the right lung. Right: Chest CT showing diffuse multifocal nodular airspace disease.

**Figure 2:**
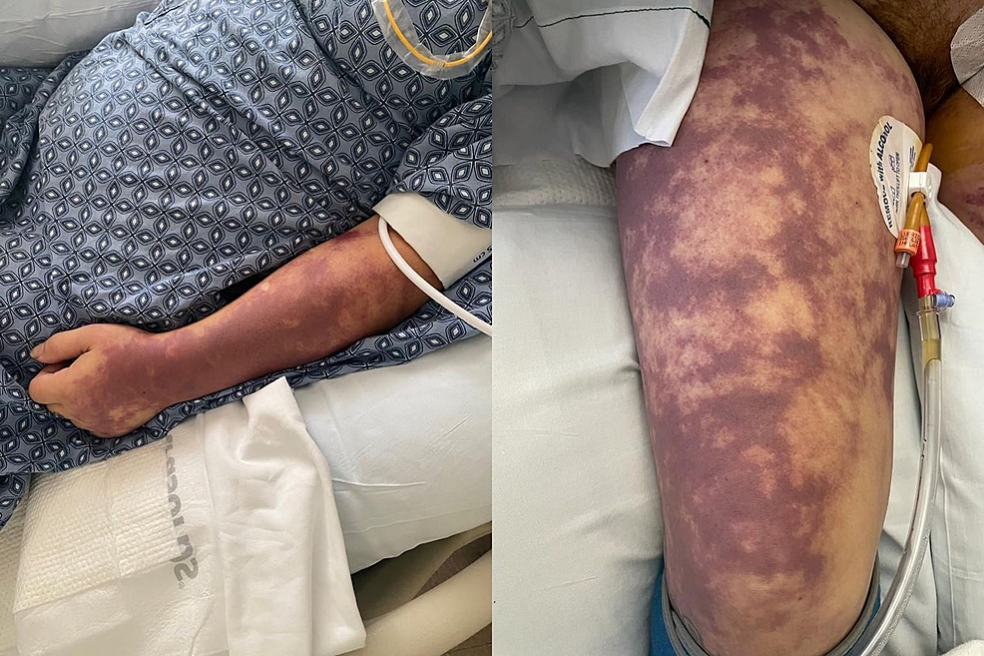
Rash. Left: Purpuric rash on upper arm. Right: Confluent mottling purpuric rash on lower extremities.

**Figure 3:**
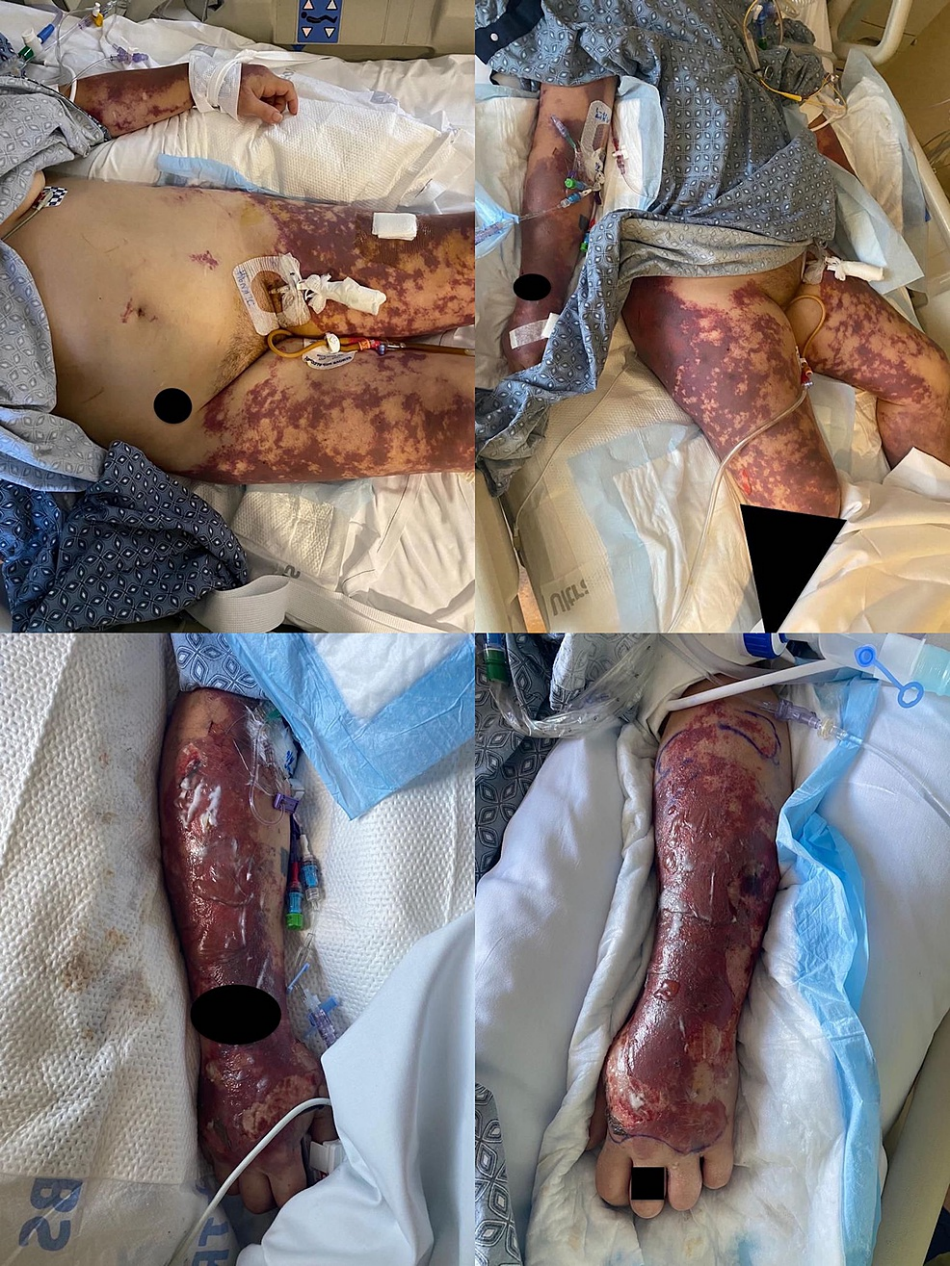
Evolving necrotizing rash with bullas. Top left: Evolving purpuric rash on lower extremities. Top right: Purpura with extensive cutaneous necrosis. Bottom left: Hemorrhagic bullas developing on upper extremities. Bottom right: Hemorrhagic blisters with retiform purpura on upper arms.

**Figure 4:**
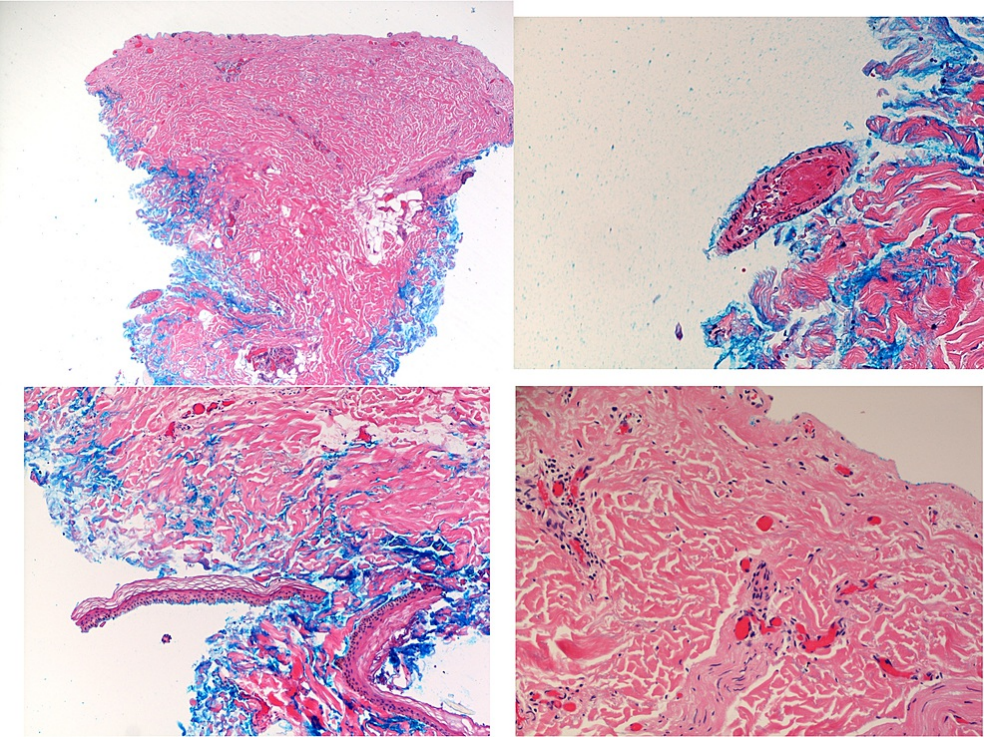
Biopsy of the involved skin tissue. Top left: Gross specimen of the biopsied tissue; deep aspect inked in blue. Top right: Skin biopsy demonstrating intraluminal fibrin deposit in a deeper dermal mid-sized vessel. Bottom left: Picture showing some epidermis that detached during the biopsy procedure. No necrosis that would point toward toxic epidermal necrolysis or Steven-Johnson syndrome could be appreciated. Bottom right: Slide exhibiting thrombi within vessels.

A 39-year-old female with a past medical history of active polysubstance abuse (alcohol, heroin, fentanyl, and amphetamine), presented to the emergency department (ED) complaining of shortness of breath and productive cough of yellow sputum of one-week duration. Moreover, she has been complaining of body aches, fevers, and generalized weakness for about a week. At the time of admission, the patient was very agitated and altered. The patient was not vaccinated against COVID-19. Infectious contacts were unknowns. She was a 25 pack-years smoker. She initially presented with a blood pressure of 64/45 mmHg, unresponsive to fluid resuscitation, and unable to keep oxygen saturation above 80% on a nonrebreather mask. The patient ended up intubated on day one for acute hypoxic respiratory failure and for being unable to protect her airway. Furthermore, norepinephrine and vasopressin drips were started to support blood pressure. She was initially diagnosed with distributive/septic shock. Initial blood work showed a WBC count of 0.9 × 10 n^3^ µL with bandemia of 12% and an absolute neutrophil count of 0.4 × 10^3^ µL. Nasopharyngeal SARS-CoV-2 polymerase chain reaction (PCR) was positive. Initial blood cultures were positive for *Streptococcus pneumoniae*. Multiple sputum cultures, as well as bronchoalveolar lavage (BAL) cultures, were positive for *Candida albicans*, *Haemophilus influenza*, and *Streptococcus pneumoniae*. Urine pneumococcal antigen was positive too. She was colonized with Methicillin-resistant Staphylococcus aureus (MRSA) in the nares. The BAL pathology results showed necrotizing inflammatory cells consistent with the diagnosis of necrotizing pneumonia (Figure 1). A Swan-Ganz catheter was placed in order to differentiate shock etiology: initial read showed cardiac output (CO) of 10.7 L/min, pulmonary capillary wedge pressure (PCWP) of 15 mmHg (N: 4-12 mmHg), and systemic vascular resistance (SVR) of 723 dyne·sec·cm^-5^ (N: 700-1,600 dyne·sec·cm^-5^, while on pressors. While in the ICU, the patient was on broad-spectrum antibiotics. The patient was positive for COVID-19, but the only treatment used included high-dose steroid with dexamethasone 6 mg for 10 days. CT scan of the chest presented diffuse multifocal nodular airspace disease as shown in Figure 1. Given that her renal function and liver function tests were compromised, the use of remdesivir was declined, and so was the use of tocilizumab given the high risk for superimposed bacterial infections. Procalcitonin and C-reactive protein (CRP) peaked at 685 ng/mL and 325 mg/L, respectively, on day three of hospital stay. On the same day, the patient developed an extensive mottling purpuric rash in the lower legs as well as distal upper extremities (Figures 2, 3). On day five of hospitalization, her need for vasopressor support decreased; however, her renal function was deteriorating to the point that she required dialysis. Over the course of the following week, the rash migrated proximally, with bullae developing over the superficial skin as well as subcutaneous crepitus (Figure 3). Furthermore, her lower extremities started to develop ischemic changes. She had skin sloughing over the upper extremities and lower extremities from the upper thighs down. There was severe cyanosis in the lower legs and very cold feet bilaterally. There were no palpable pulses. Heparin drip was initiated; however, it was later stopped because the patient had frank bleeding from the endotracheal tube, IV sites, vulva, and gastrointestinal tract requiring multiple blood transfusions. A biopsy of the involved tissue was performed confirming the diagnosis of purpura fulminans (PF) (Figure 4). Other labs values were obtained to support a diagnosis of PF and rule out other underlying pathologies as shown in (Table 1).

**Table 1:** Significant labs values obtained from the patient. ADAMTS13: a disintegrin and metalloproteinase with a thrombospondin type 1 motif, member 13,  ANA: antinuclear antibodies.

	Day 3	Day 4	Day 6	Day 10
Protein C [70%-180%]	20 L	11 L		84 L
Protein S [60%-140%]		<10 L		91 L
ADAMTS13 [0.68-1.63 IU/mL]	0.5 L			
D-dimers [0.19-0.52 mg/L]	7.42 mg/L	6.29 mg/L		14.22 mg/L
Antithrombin-3 activity [80%-135%]		51 L		
Factor II [70%-150%]				77
Factor X activity [70%-150%]				118
Hepatitis A IgM AB	Nonreactive			
Hepatitis Bs antigen	Nonreactive			
Hepatitis B core IgM Ab	Nonreactive			
Hepatitis C antibody	Nonreactive			
HIV viral load (cps/mL)	Nonreactive			
HIV viral load log	Nonreactive			
IgG [635-1741 mg/dL]			749 mg/dL	
IgA [66-436 mg/dL]			189 mg/dL	
IgM [43-279 mg/dl]			131 mg/dL	
ANA			Negative	
Proteinase 3 (PR3)			No antibody detected	
Myeloperoxidase Ab			No antibody detected	
Cardiolipin IgG Ab			No antibody detected	
Cardiolipin IgM Ab			No antibody detected	
Complement C3 [79-152 mg/dL]			86 L	
Complement C4 [18-55 mg/dL]			13 L	

The patient expired on day 19 of hospital stay after her family members decided to withdraw life-supporting measures given her overall deteriorating clinical status.

## Discussion

Purpura fulminans is a rare presentation of a thrombotic disorder that rapidly results in tissue ischemia with skin necrosis together with disseminated intravascular coagulation (DIC). The etiology can be multifactorial; however, in our case, the culprit could reside in the streptococcal bacterial infection leading to sepsis. Exact pathogenesis of streptococcal purpura fulminans is not clearly understood; however, streptococcal pyrogenic exotoxins (speA, speB, and speC) are known to induce cytotoxicity and pyrogenicity leading to a cytokine storm that can rapidly progress to septic shock, DIC, and multiorgan failure [7]. On the other hand, COVID-19 also induces a state of profound inflammatory response from cytokine storm, endothelial damage, and microvascular thrombosis. Mainstay of treatment includes addressing the underlying causative etiology and administration of activated protein C (APC) or fresh frozen plasma (FFP) [8]. Prognosis is poor, and without treatment, soft tissue gangrene can lead to loss of limb due to ischemia. Microvascular thrombosis and hemorrhagic ischemia can affect multiple tissues such as lungs, kidneys, and central nervous system resulting in multiorgan failure with high mortality and long-term morbidity in survivors. Unfortunately, our patient deteriorated rapidly, with gangrenous changes on distal limbs toward the end of her stay. Lack of Xigris (recombinant form of activated protein C) availability made the caring for this patient more challenging. Interestingly, while the PROWESS trial was successful in demonstrating an improvement in mortality in patients with severe sepsis receiving APC, subsequent studies including PROWESS-SHOCK trial failed to demonstrate a mortality improvement [8]. More recently, a mutant form of APC, called 3K3A‐APC, has a decreased affinity for its substrate factor Va, therefore decreasing the risk of bleeding but retaining its beneficial effect of anti‐inflammatory and cytoprotective cell‐signaling properties through the interaction with the endothelial protein C receptor (EPCR) and protease-activated receptor 1 (PAR-1). Further animal studies have shown beneficial properties of APC and 3K3A-APC by reducing organ dysfunction [9]. One study was significant at showing reduction of lung damage and cytokine storm after one bolus injection of APC in rats models [9]. Early clinical trials, including the RHAPSODY trial, demonstrated the safety of the molecule 3K3A-APC in healthy human subjects with a maximal tolerated dose of 540 μg/kg [10-11]. We are hopeful that these preclinical trials may be a trampoline for the use of APC in reducing SARS-CoV-2-induced lung damage in selected patients with COVID-19 who are critically ill, since it would reduce overall uncontrolled inflammation and endotheliitis as well as attenuation of ischemia-reperfusion injury in lungs and other organs.

## Conclusions

COVID-19 disease can most often present as a respiratory illness; however, recent data have shown that it is associated with arterial and venous thrombosis leading to serious adverse events and possibly death. Anticoagulation alone may not be enough in preventing such consequences. As in our case report, our patient deteriorated quickly and ended up with gangrenous changes in her extremities and a plan for amputation if she had survived. This microangiopathy might have resulted from microthrombosis related to COVID-19 or DIC. Many ongoing researches are investigating the use of coagulation inhibitors such as antithrombin and activated protein C in critically ill SARS-CoV-2 patients. Hopefully, these trials will provide definitive strategies to prevent these patients from worse outcomes.
